# Long working hours, poor sleep quality, and work-family conflict: determinant factors of fatigue among Indonesian tugboat crewmembers

**DOI:** 10.1186/s12889-021-11883-6

**Published:** 2021-10-09

**Authors:** Muchtaruddin Mansyur, Risna Sagitasari, Grace Wangge, Astrid B. Sulistomo, Aria Kekalih

**Affiliations:** 1 Occupational Medicine Division, Community Medicine Department, Faculty of Medicine Universitas Indonesia, Jakarta, Indonesia; 2 South East Asian Ministers Education Organization, Regional Center for Food and Nutrition/SEAMEO-RECFON, Pusat Kajian Gizi Regional Universitas Indonesia, Jakarta, Indonesia; 3 Occupational Medicine Post Graduate Study Program, Community Medicine Department, Faculty of Medicine Universitas Indonesia, Jakarta, Indonesia

**Keywords:** Fatigue, Tugboat crewmembers, Seafarer, Working hours, Sleep quality, Work-family conflict

## Abstract

**Background:**

Tugboat crewmembers are susceptible to fatigue during their 24-h work shifts, despite the availability of rest time. The fatigue experienced by seafarers contributes to marine accidents and metabolic and cardiovascular diseases, which have long-term effects. This study aimed to analyse the association between working hours and fatigue and other possibly related factors in tugboat crewmembers.

**Method:**

This comparative cross-sectional study included 127 tugboat crew members from 15 randomly chosen tugboats in Samarinda Harbor, Indonesia. Their fatigue levels while at work were measured using a reaction timer and standardised questionnaire. Personal and occupational data of crewmembers, including age, marital status, rating (job ranking), duration on board, length of seafaring experience, watch system, smoking status, coffee and alcohol consumption, and working hours, were collected. Moreover, sleep quality and stress levels related to work-family conflict were measured and analysed using the Pittsburgh Sleep Quality Index (PSQI) and Work-Family Conflict Scale (WCFS), respectively.

**Results:**

The study found that 40.2% of the subjects were classified as having fatigue. The determinant factors were long working hours (> 72 h/week), poor sleep quality, and work-family conflict [adj. OR = 13.32; 95%-CI (4.78–31.23)] and *p* < 0.001, [adj. OR = 4.49 (1.39–14.52)] and *p* = 0.012, [adj. OR = 2.87 (1.12–7.33)] and *p* = 0.028, respectively. However, personal and occupational factors, including age, marital status, duration on board, length of seafaring experience, smoking status, and coffee and alcohol consumption, were not significantly associated with crewmember fatigue.

**Conclusion:**

The incidence of fatigue among Indonesian tugboat crewmembers operating on the Mahakam River was considerably high. Working hours, sleep quality, and work-family conflict were strongly associated with fatigue in tugboat crewmembers; therefore, the working hours of tugboat crewmembers need to be improved. Crewmember lifestyle variables need to be studied further.

## Background

Fatigue is characterized by a reduction in physical and mental capability resulting from physical, mental, or emotional exertion. It may impair nearly all physical abilities, including strength, coordination, decision-making, balance, speed, and reaction time. Fatigue occurs due to an imbalance between physical and mental strength during activities and helps the body and brain recover after activities [1]. Fatigue is experienced frequently among the general working population, with the estimated prevalence being as high as 22% [2]. Working at sea certainly makes one susceptible to fatigue; therefore, the shipping industry is increasingly concerned about the possible effects of fatigue on personal and operational safety as well as workers’ general health and wellbeing [2, 3]. Roberts et al. [4] reported that although the fatal accident rate in the British shipping industry has continued to decrease in recent years, the incidence rate in Great Britain was higher than those among seafarers employed by merchant fleets worldwide. Maritime incidence analyses have shown that approximately 80% of maritime casualties are due to human error [4, 5], and 26% of shipping incidents are associated with fatigue in seafarers [6, 7]. Moreover, fatigue, especially chronic fatigue, plays a role in the development of metabolic disorders and cardiovascular diseases. Low-grade chronic inflammation due to chronic fatigue has long-term effects that cause metabolic and endocrine disorders, cardiovascular diseases, autoimmune diseases, and depression [8, 9].

Watchkeeping and short port turnaround times often increase the frequency of activities, resulting in long and irregular working hours [10]. The International Labor Organization and International Maritime Organization (IMO) have established mandatory provisions outlined in the Standards of Training, Certification, and Watchkeeping (STCW), in which the maximum number of working hours for seafarers is 14 h per day or 72 h per 7 days [1, 11]. The International Federation of Transport has reported that 25% of seafarers suffer from fatigue because they work for more than 80 h per week [12].

The watch officer’s sleeping time depends mainly on the type of watch system implemented. Two fixed watch systems are usually applied: the 4/8 watch system (4 h work and 8 h rest) and the 6/6 watch system (6 h work and 6 h rest). Seafarers are more likely to tire during a shift under the 6/6 watch system than under the 4/8 watch system, especially those with early morning shifts [13]. Seafarers who work under the 4/8 watch system have better sleep efficiency, more continuous sleep, and a larger number of sleep hours than those under the 6/6 watch system [14]. The risk of fatigue also depends on the seafarer’s rating, as different levels have different job demands, causing varying fatigue levels. For example, officers may experience high stress levels due to their comprehensive personnel and material responsibilities. However, non-officers face other risks, as they are exposed to strenuous physical activities and job demands [15]. Regarding the length of service, fatigue is associated with the pressure to work continuously, an increase in the number of jobs, and adaptation to new environments. A shorter duration onboard was associated with a higher risk of fatigue than a longer duration onboard [16]. Dohrmann et al. [17] found that fatigue among ferry shipping crewmembers was associated with work-family conflict and poor supervisor support. In their study, these two factors were significantly associated with two subdimensions of fatigue, physical exertion and physical discomfort.

However, Hystad and Eid [16] found no linear relationship between duration at sea and fatigue.

Additional factors contributing to fatigue are irreversible, job-related, physical factors, such as noise and vibration during working hours and leisure time, which decrease the restorative effect during free time [15].

Marine sector activities, environments and vehicle types, such as cruise ships, ferries, floating cranes, tankers and other sea and river transportation, are associated with various health and safety risks. For example, the high density of cruise ships in the path of tugboats and the river’s geography, with a winding path and varying depths, increases the risk of accidents on the Mahakam River, Indonesia. In 2017, the Samarinda harbourmaster recorded 13 maritime incidents involving tugboats in the Mahakam River; the incidents ranged from grounding to ship burning and collision. The high incidence of accidents and the lack of investigation into whether these accidents were related to human factors, such as fatigue, warrant a preliminary study to determine factors associated with fatigue among tugboat crewmembers.

## Methods

### Study design

This comparative cross-sectional study aimed to identify factors associated with fatigue in seafarers. This study took place in Samarinda Harbor, which hosts vessels from various shipping companies, in Samarinda city from May to June 2018. Tugboat crewmembers were the subjects of this study. The purpose of this study was discussed with all the shipping companies prior to the initiation of the study. Tugboats were selected in cooperation with the companies based on their availability considering their voyage schedule. Only ships with a 7-day minimum voyage duration were included and randomised. Assuming there were 8–10 crewmembers per tugboat and considering a minimum sampling size of 126 subjects, researchers included all crewmembers from the 15 randomised tugboats for a total of 138 respondents.

### Data collection

Data collection consisted of two steps. First, before sailing, the respondents were provided with a log sheet to record work-rest time during their voyage. Researchers waited until all the respondents returned from a minimum 7-day voyage. A total of 127 respondents returned from sailing. Second, demographic data, such as age, marital status, smoking status, caffeine and alcohol intake, and work-related data, such as rating, watch system, length of seafaring experience, and duration on board, were collected. On the questionnaire, the rating (officer or non-officer), the watch system (4/8 or 6/6), the length of seafaring experience and the duration on board were open-ended questions. Those who worked under a watch system other than the 4/8 or 6/6 system were excluded from the data analysis. Total working hours per week was obtained from the log sheet of work-rest time. The voyage memo was used to cross-check the log sheet data to limit recall bias and ensure data accuracy. Subjective sleep quality data were assessed using the Pittsburgh Sleep Quality Index (PSQI), which consists of a self-rated questionnaire with seven components: subjective sleep quality, sleep latency, sleep duration, sleep efficiency, sleep disturbance, use of sleep medication, and daytime dysfunction. Scores higher than 5 points indicate poor sleep quality. The PSQI was introduced by Buysse et al. [18] and validated in the Indonesian language, with satisfactory validity and reliability [19, 20].

Work-family conflict was evaluated using the Carlson et al. [21] tool of Work-Family Conflict Scale (WFCS) that has been validated in the Indonesian version [22]. The Work-Family Conflict Scale with higher scores indicating more conflict. In this study, the conflict score was categorised as “yes” or “no”, and the median value of the scale was used to determine the cut-off point for categorisation.

Fatigue level was determined using a combination of the reaction time test and the fatigue questionnaire. The reaction time test used was the L77/Lakassidaya-type reaction timer, developed and validated in Indonesia [23] This tool is portable, so the reaction time measurement can be done on the tugboat when the respondent returns from a minimum 7-day trip. The assessment is intended to represent the reaction time at the end of the crewmembers’ duty schedule; therefore, measurements were taken during their working time before disembarking. The ensure that the study subjects were comfortable and focused on the assessment, all crews were still in service but free from assignments during measurement.

The crew was informed about the measurement schedule beforehand. The reaction time was calculated based on the middle 10 measurements of 20 total measurements. The first five and last five measurements were excluded because the former were taken in the adaptation stage, and the latter were taken in the boredom stage.

The fatigue questionnaire is a standardised instrument for the subjective measurement of fatigue at work adopted from the Japan Industrial Fatigue Research Committee and previously validated in the Indonesian language, with satisfactory validity and reliability [24]. Participants were asked to perceive how they felt and then answer 17 questions on a 3-point Likert scale, with a score of 0 representing “Never;” a score of 1 representing “Yes, Seldom;” and a score of 2 representing “Yes, Often.” The cumulative score range was 0 to 34. The questionnaire classified subjects as feeling fatigued if they had a cumulative score of more than 17. To enhance the fatigue assessment instrument’s specificity, fatigue was determined if the study subjects had both a reaction time longer than 240,000 milliseconds [23] and a fatigue questionnaire score above 17 points.

The participants’ daily routines were not altered to control for objective and subjective measurement bias. The participants provided informed consent before participating in the study and were assured that their answers were confidential. The Research Ethics Committee of the Faculty of Medicine, Universitas Indonesia, approved this study under protocol no. 18-05-0563 and approval reference no. 0907/UN2.F1/ETIK/2018.

### Statistical analysis

Data were analysed using SPSS for Windows (version 20.0). Continuous variables, such as the length of seafaring experience and duration on board, are expressed as means/medians (*+*standard deviations/min-max), and median values were used as cut-off points to categorise the varying outcomes. Fatigue level was the dependent variable, while the others were independent variables. Categorical independent variables were analysed using the Chi-Square test, except for age, which was a continuous variable, and nonnormally distributed data were analysed using the Mann-Whitney test. Variables with a *p*-value < 0.1 in the bivariate analysis were included as candidate variables in the multivariate analysis [25].

Multinomial logistic regression analysis was performed to obtain the adjusted odds ratios for the determinant factors. However, the dataset was not large enough (*N* = 127) to include more candidate variables; the data analysis was limited to four variables to meet a minimum of at least five multinomial events per variable (EPVm) [26].

## Results

Among the 138 respondents selected by sampling, nine crewmembers from 1 tugboat had a prolonged voyage, making it impossible for them to continue participation in this study. Moreover, one respondent could not continue participation due to sickness while on board, and one participant had to leave the ship for family reasons; hence, these participants were excluded from the study analysis. In total, 127 participants, including 57 respondents who worked more than 72 h per week and 70 respondents who worked less than 72 h per week, were included. The crewmembers’ ages ranged from 21 to 60 years. More than half of them were not married (51.2%), were smokers (63%) and consumed coffee (53.5%) and/or alcohol (22.8%). More than 75% of the respondents had poor sleep quality; 54.3% received less than 7 h of sleep per day, and more than 50% experienced work-family conflict.

The study sample comprised 63 officers (49.6%) and 64 non-officers (50.4%), with the majority of respondents under the 4/8 watch system (82.7%). Their length of seafaring experience was ranged from 1 to 35 years, with 55.9% of the respondents having less than 6 years of seafaring experience. Approximately 51.2% of the respondents had a duration of less than 6 months on board. This study also found that 44.9% of the respondents worked more than 72 h/week.

The measurement results showed that 40.2% of the tugboat crewmembers experienced fatigue. Of the 59.8% that did not experience fatigue, 45.7% had a prolonged reaction time without an increased score on the subjective fatigue questionnaire.

Table 1 summarises the results of the bivariate analyses and four variables with a *p*-value < 0.01: sleep quality, work-family conflict, length of seafaring experience, and working hours.

**Table 1:** Association of tugboat crewmember characteristics with fatigue categories

Variable	Fatigue; f (%)		*p*-value
	Yes	No	
Age (median; min-max. in years)	28; 20–61	29; 20–56	0.76^*^
Marital status			
- No	30 (46.2%)	35 (53.8%)	0.16^**^
- Yes	21 (33.9%)	41 (66.1%)	
Smoking status			
- Medium smoker	9 (42.9%)	12 (57.1%)	0.60^**^
- Light smoker	25 (42.4%)	34 (57.6%)	0.52^**^
- Non-smoker	17 (36.2%)	30 (63.8%)	Reference
Caffeine consumption			
- > 2 cups/day	5 (35.7%)	9 (64.3%)	1.00^***^
- 1–2 cups/day	25 (46.3%)	29 (35.7%)	0.25^**^
- None	21 (35.9%)	38 (64.4%)	Reference
Alcohol consumption			
- Yes	17 (58.6%)	12 (41.4%)	0.02^**^
- No	34 (34.7%)	64 (65.3%)	
Sleep quality			
- Poor	45 (46.9%)	51 (53.1%)	0.01^**^
- Good	6 (19.4%)	25 (80.6%)	
Work-Family Conflict			
- Yes	35 (54.7%)	29 (45.3%)	< 0.01^**^
- No	16 (25.4%)	47 (74.6%)	
Watch system			
- 6/6	9 (40.9%)	13 (59.1%)	0.94 ^**^
- 4/8	42 (40.0%)	63 (60%)	
Rating			
- Officer	20 (31.7%)	43 (68.3%)	0.06 ^**^
- Non-Officer	31 (48.4%)	33 (51.6%)	
Duration on board			
- < 6 months	28 (43.1%)	37 (56.9%)	0.49^**^
- > 6 months	23 (37.1%)	39 (62.9%)	
Length of seafaring experience			
- < 6 years	36 (50.7%)	35 (49.3%)	0.01^**^
- > 6 years	15 (26.8%)	41 (73.2%)	
Working hours			
- > 72 h/week	40 (65.3%)	17 (34.7%)	< 0.01^**^
- < 72 h/week	11 (24.4%)	59 (75.6%)	

* = Mann-Whitney test

** = χ2 test

*** = Fisher’s exact test

f (%) = Frequency (percentage)

The following candidate variables that had a *p*-value < 0.01 in the bivariate analysis were included in the multivariate analysis: working hours, sleep quality, work-family conflict, and length of seafaring experience.

Table 2 presents the multivariate analysis results. Working hours more than 72 h/week was strongly associated with fatigue (*p* = < 0.001, adj. OR = 13.32, 95% CI; 4.78–31.23), and a significant association between fatigue and sleep quality (*p* = 0.012, adj. OR = 4.49, 95% CI; 1.39–14.52) was noted. In addition, participants with work-family conflict had higher odds of fatigue than those without work-family conflict (*p* = 0.028, adj. OR = 2.87, 95% CI; 1.12–7.33).

**Table 2:** The determinant factors of fatigue among seafarers

Variable	Fatigue; f (%)		*p*-value	Adj. OR [95% CI]
	Yes	No		
Working hours			< 0.001	13.32 [4.78–31.23]
- > 72 h/week	40 (65.3%)	17 (34.7%)		
< 72 h/week	11 (24.4%)	59 (75.6%)		
Sleep quality			0.012	4.49 [1.39–14.52]
- Poor	45 (46.9%)	51 (53.1%)		
- Good	6 (19.4%)	25 (80.6%)		
Work-family conflict			0.028	2.87 [1.12–7.33]
- Yes	35 (54.7%)	29 (45.3%)		
- No	16 (25.4%)	47 (74.6%)		

Goodness of Fit = 0.43

R^2^ Nagelkerke = 0.49

*Adj. OR* Adjusted odds ratio, adjusted for length of seafaring experience

In the bivariate analysis, the length of seafaring experience was significantly associated with crewmember fatigue. However, after adjustment for the three main variables above, the multivariate analysis found that the length of seafaring experience (*p* = 0.154) was not significantly associated with fatigue.

## Discussion

This study found that the fatigue prevalence among tugboat crewmembers in Samarinda Harbor was 40.2%. The related factors were long working hours (particularly those exceeding 72 h/week), sleep quality, and work-family conflict.

The prevalence of fatigue among the subjects in this study was slightly lower than the prevalence among floating crane ship crewmembers reported in a previous study (69.4%) [27]. This difference might be due to variations in the work characteristics between these two ship types. Unlike a tugboat, a floating crane generally stays in one place for an extended period because of the work nature. Lack of an opportunity to exit the ship to prevent boredom induces a monotonous working rhythm [16]. On a tugboat, even though the total sailing time may be longer, during times when the ship is loading or unloading, the crew may disembark the boat to shop for food and supplies or for entertainment. It has been shown that boredom effects contributed the most to the performance of crews [28].

This study found that working hours were significantly associated with fatigue [adj. OR = 13.32; 95% CI (4.78–31.23)]. More than 40% of the study subjects worked more than 72 h/week, exceeding the seafarer working hour limit established by the STCW-IMO 2019 regulation [1]. This study supports the finding of Salyga [29], who studied passenger ship crewmembers. Generally, passenger ships use automated systems in their daily operation to minimise fatigue experienced by seafarers.

This study showed that 75.6% of the respondents had poor sleep quality, which significantly increased the likelihood of fatigue [adj. OR = 4.49; 95% CI (1.39–14.52)]. A similar finding was reported by Andhika [27], who found that respondents with poor sleep quality had a significant chance of experiencing fatigue. Smith et al. [15] also found that poor sleep quality was significantly related to fatigue. Poor sleep quality contributes to fatigue due to insufficient relief from acute fatigue, and cumulative fatigue eventually progressed to chronic fatigue. Wang [30] and Jepsen [31] have proposed that chronic fatigue is due to the accumulation or recurrence of acute fatigue. Watchkeeping duty, especially at night, disturbs sleep, making it challenging to maintain good sleep quality [17]. In addition, Smith and McNamara [2] found that sleep quality was affected by ship noise and movement and reported that 44% of respondents considered noise a nuisance.

Similar to Dohrmann et al.’s study [32], this study found that work-family conflict was significantly associated with tugboat crewmember fatigue [Adj. OR = 2.87: 95% CI (1.12–7.33)]. Further, their study explained that work-family conflict contributed to a heightened risk of physical exertion and physical discomfort in the physical subdimensions of fatigue. Sampson & Ellis [33] identified that the potential main factors related to family were missing family members and receiving bad news from home. To prevent mental health issues among seafarers, they recommended the availability of confidential counselling services.

Smith [12] stated that several job conditions, including night shift work, may cause acute and chronic fatigue, decrease awareness and increase sleep debt. Moreover, Lacourt et al. [8] found that working conditions, including night shift work, changed circadian rhythms, increasing persistent fatigue associated with chronic low-grade inflammation. Furthermore, Chen et al. [9] noted that chronic low-grade inflammation led to various prolonged stress-related disorders, such as cardiovascular disease, diabetes, autoimmune conditions, and even clinical depression.

The results of the multivariate analysis did not reveal a relationship between the length of experience of seafarers and the incidence of fatigue in tugboat crewmembers. This result is similar to that of Hystad’s [16] systematic review that found no relationship between the length of experience of seafarers and fatigue in crew members on various types of vessels. They argued that seafarers’ psychological capacity in response to environmental stressors is necessary to cope with the stressful onboard environment.

This study found a nonsignificant association between job rating and fatigue and that non-officers had the same likelihood of fatigue as officers. However, many studies have found different results. Andhika’s study [27] found that non-officers experienced more fatigue on floating cranes than officers. Smith et al. [12] also found that non-officers had a higher workload because of their function as technical operators and carried out more strenuous physical activities with longer working hours than officers. Burke [34] found that officers had relatively more regular shifts and working hours than non-officers. Therefore, the likelihood of fatigue in non-officers was higher than that in officers [10, 35] These different study results regarding the relationship between job rating and fatigue may be explained by the fact that the tugboats had fewer crew members than the other types of ships in other studies. The small number of tugboat crewmembers makes the workload similar between job ratings, both quantitatively and qualitatively.

This study has some limitations. One of the limitations is the common method bias/variance that was indicated by the final model coefficient determinant, which was not high enough. This study’s potential common method bias might be due to limited candidate variables included in the data analysis, resulting from the bivariate analysis that considered only a *p*-value of < 0.01. Nevertheless, the sample size is sufficient to answer the research questions. The other study limitation is no direct observation during work-rest time recording.

Additionally, work-rest time data depended solely on the respondents’ answers to the self-completed questionnaire. Potential bias was minimised by cross-checking with the voyage memo. Fatigue assessment is also needed. However, as Volker et al. [36] noted, there is no existing, agreed-upon single method to detect and quantify fatigue or a robust diagnostic tool to measure subjective fatigue. Finally, this study samples was limited to tugboat crew members; nevertheless, this study’s results might be generalised to other ships’ crew members with similar personal characteristics, tasks as seafarers, and working environments.

Based on the results of the analysis, stakeholders should consider some recommendations. The high prevalence of fatigue among seafarers should be prevented and controlled by addressing the determinant factors enumerated in this study. Some unhealthy habits can aggravate fatigue, such as low-grade chronic inflammation, which increases the risk of metabolic disorders and cardiovascular diseases [9]. Therefore, workers should improve their knowledge and awareness regarding fatigue and its related factors. Crewmembers should adopt a healthy lifestyle by avoiding smoking and alcohol, consuming a balanced diet onboard, exercising regularly, and enhancing sleep quality through sleep hygiene or strategic napping.

Government agents, especially the harbourmaster, should consider the importance of monitoring working hours to maintain health and safety onboard and raising awareness among stakeholders about the dangers of fatigue in the workplace due to prolonged and irregular working hours and lack of sleep onboard. Marine companies are encouraged to conduct screening for fatigue and hold sleep disturbance management sessions in collaboration with health and safety institutions.

Marine companies should start developing fatigue and working hour monitoring systems and risk management systems as strategies to mitigate fatigue in mariners. In addition to personal psychological support, companies should promote wellness programmes for crew members onboard and enhance sleep quality by improving ship accommodation facilities and maintaining good housekeeping.

The four variables included in the multinomial regression analysis accounted for 49% (Nagelkerke R^2^ = 0.49) of fatigue, which means that further studies are needed to explore additional variables accounting for to the remaining percentage, including sub-dimensional measurements of fatigue.

## Conclusion

The prevalence of fatigue among tugboat crewmembers in Samarinda is relatively high. Fatigue was significantly associated with working hours, quality of sleep, and work-family conflict. Seafarers who worked more than 72 h per week experienced more fatigue than those who worked 72 h or less. Additionally, the odds of fatigue in workers with poor-quality sleep was more than four times higher than that in those with good sleep quality. In those with work-family conflict, the odds of fatigue was increased by approximately three times than that in those without work-family conflict. All stakeholders should fully comply with the STCW-IMO regulations regarding seafarer working hours, limiting working hours to a maximum of 14 hours per day or 72 h per 7 days. Stakeholders should also consider control measures for the risk factors identified in this study. Psychosocial support programmes should be in place to assist crewmembers with work-family conflict or other psychological problems.

Further study is necessary to strengthen prevention and control programmes targeting fatigue and its health and safety consequences among seafarers by exploring both occupational and nonoccupational risk factors.

## Data Availability

The datasets used and/or analysed during the current study are available from the corresponding author upon reasonable request.

## Abbreviations

Adj. OR: Adjusted odds ratio
IMO: International Maritime Organization
PSQI: Pittsburgh Sleep Quality Index
R^2^: Coefficient of determinant
STCW: Standards of Training, Certification, and Watchkeeping
WFCS: Work-family conflict scale
